# Bacterial Subspecies Variation and Nematode Grazing Change P Dynamics in the Wheat Rhizosphere

**DOI:** 10.3389/fmicb.2018.01990

**Published:** 2018-09-05

**Authors:** Usman Irshad, Etienne Yergeau

**Affiliations:** ^1^Centre INRS–Institut Armand-Frappier, Institut National de la Recherche Scientifique, Université du Québec, Laval, QC, Canada; ^2^Department of Environmental Sciences, COMSATS University Islamabad, Abbottabad, Pakistan

**Keywords:** phosphorus solubilizing bacteria, *Pseudomonas poae*, nematodes, *phnX* gene, wheat rhizosphere, tricalcium P

## Abstract

Low phosphorus soils are thought to constitute the majority of soils worldwide and cannot support intensive agriculture without high fertilizer inputs. Rhizobacteria are well-known to modify P dynamics and an increased bacterial diversity normally has a positive impact on various process rates. However, it is not known how variation in bacterial diversity at the subspecies level could influence trophic interactions in the rhizosphere and its consequences on plant P nutrition. We therefore hypothesized that the interactions between closely related P solubilizing bacteria and their grazing nematodes could improve plant P dynamics from an unavailable P source. We isolated four *Pseudomonas poae* strains and extracted nematodes from a Saskatchewan wheat field soil sample. The potential of all bacterial isolates with and without nematodes for increasing P availability in the wheat rhizosphere was tested in controlled microcosms with Ca_3_(PO_4_)_2_ as sole P source. Liberated P, phosphatase activity, plant P and bacterial abundance based on *phnX* gene copies were determined. Phosphorus solubilization efficiency of isolates varied between isolates whereas phosphatase enzyme activity was only detected under nematodes grazing and during the first 15 days of the experiment. Nematodes grazing upon individual *Pseudomonas poae* increased phosphatase enzyme activity, bacterial abundance, but decreased plant P concentration compared to non-grazed system. In contrast, the treatment combining all *Pseudomonas poae* isolates together with nematodes resulted in significant increases in P availability and plant P concentration. Diverse P-solubilizing efficiency and interaction with nematodes within the same bacterial “species” suggest that P dynamics might be linked to micro variation in soil diversity that would not accurately be picked up using common tools such as 16S rRNA gene sequencing.

## Introduction

Low phosphorus soils are thought to constitute the majority of soils worldwide. Soil P consists of inorganic forms, such as rock phosphate and organic forms derived from plants, animals and microbial biomass decay (Behera et al., 2014). Even when the phosphorus inputs are very high, less than 5% of the soil P is bioavailable to plants (Brady and Weil, 2008). Since up to 40% of cultivable microbes can solubilize inorganic P and release plant utilizable forms directly into solution (Nazir et al., 2017), efforts to improve efficiency of P use are focused on microorganisms. Many P-solubilizing bacteria are found in the *Pseudomonas* and *Bacillus* genera (Illmer and Schinner, 1992; Irshad et al., 2011), and they generally mineralize/solubilize P either by secreting organic acids or phosphatases (Gugi et al., 1991; Lemanowicz, 2011). The capacity of *Pseudomonas* to transform P into bioavailable forms through mineralization or solubilization varies depending on the species and substrates. For instance, *Pseudomonas fluorescens* can use acid phosphatase on non-specific substrates (Gugi et al., 1991). In liquid culture, different *Pseudomonas* species can solubilize from 52 to 156 mgL^−1^ Ca_3_(PO_4_)_2_ (Illmer and Schinner, 1992). It has also been previously shown that closely related isolates, often from the exact same bacterial species, differed significantly in their capacity to solubilize P (Zabihi et al., 2011; Singh et al., 2014; Jiang et al., 2017).

The degree of P-solubilization by bacteria observed *in vitro* often differs when bacteria are grown in association with plants. When in a mutualistic association with plants, P-solubilizing microbes provide soluble P in exchange for plant carbon (Becquer et al., 2014). Prey–predator interactions are also known to interactively determine the release of nutrients (Saleem et al., 2016). In addition, interactions of bacteria with each other and with grazers have been observed in the rhizosphere. Predation of bacteria significantly changes plant growth via changes in bioavailable N and P dynamics. Rosenberg et al. (2009) observed positive effects of bacterial grazing amoeba on *Arabidopsis* plant growth, suggesting an important role for bacterial predation in structuring bacteria–plant interactions.

Bashan et al. (1995) showed that when soil nutrients are exhausted, the abiotic soil parameters together with the biological interactions in the rhizosphere, determine the survival rate of inoculated bacteria in soil. The interaction of bacteria with their microbial food web partners, such as protists and nematodes, determines their nutrient release capacity. Among these, soil bacterivorous nematodes are probably one of the most crucial factors in determining the effectiveness of P solubilizers (Chen et al., 2007; Irshad et al., 2012; Becquer et al., 2014).

Recently, Cheng et al. (2016) showed that *Pseudomonas*-feeding nematodes influence soil nutritional status by significantly influencing nitrogen levels. They argued that nematodes grazing of bacteria can accelerate the turnover of nutrients assimilated in bacterial biomass. Khan and Joergensen (2009) showed that phosphorus solubilizing bacteria alone are not sufficient as biofertilizer because bacteria assimilate soluble P as well, making it unavailable to plants. Irshad et al. (2012) showed highly significant effect of bacterial grazing on the P release from an organic sodium phytate source. Irshad et al. (2011, 2012, 2013) further showed the importance of bacteria grazing nematodes during plant N and P acquisition, but these studies were limited to pine seedlings. While many studies showed the positive effect of bacteria feeding nematodes for plant N nutrition (Villenave et al., 2004; Bonkowski et al., 2009; Cheng et al., 2016; Zhang et al., 2016), little is known about the contribution of nematodes to the release of P from microbial biomass (Marschner et al., 2011), the influence of microbial microdiversity on P-solubilization and the variation in the capacity to interact with nematodes during P-solubilization between very closely related isolates of same bacterial species. We hypothesized that even closely related isolates would differ in their capacity to solubilize P and to interact with nematodes and that, when combined, the different isolates would be more efficient to improve plant P uptake than alone. Therefore, the aim of our study was to evaluate the prey–predator interactions of four closely related *Pseudomonas poae* isolates with their feeding nematodes in the wheat rhizosphere and the consequences on the ability to mobilize inorganic P from tricalcium phosphorus. We measured the liberated P, plant accumulated P, phosphatase activity, abundance of bacterial isolates and nematodes in the rhizosphere of wheat growing in controlled microcosms.

## Materials and Methods

### Isolation and Characterization of Phosphorus Solubilizing Bacteria

Soil samples were randomly taken from a Saskatchewan wheat field at a depth of 6 to 12 cm. A composite sample was prepared and used for isolation of bacteria and nematodes. Dilution plate technique was used to isolate bacteria from rhizospheric soil. Soil suspensions from 10^−2^ to 10^−4^ dilutions were plated on solid pure Pikovskaya’s medium (Nautiyal, 1999) with TCP (Tricalcium Phosphate) as the sole P source. This medium was used because of its specificity for the isolation of efficient phosphorus solubilizing bacteria (Nautiyal, 1999) and because TCP it is most abundant form of P present in alkaline soils where solution P immediately converts into fixed P by binding with calcium. The concentration of TCP was adjusted according to previous reports (Irshad et al., 2011; Nazir et al., 2017). Plates were incubated for 48 h at 30°C and 20 bacterial colonies were able to solubilize TCP. Among them, four efficient phosphorus solubilizing isolates were selected on the basis of free orthophosphate liberation in medium. Solubilization efficiency was calculated as described by Zakry et al. (2010) by using growth of bacterial colony according to following formula.

P-solubilization efficiency = solubilization diameter/growth diameter × 100.

Selected isolates were then maintained on solid Pikovskaya’s medium with TCP. Their ability to grow and to liberate free phosphate in liquid Pikovskaya’s medium with TCP (same composition as above without agar) was further studied. The identification of the isolates was carried out as described by Nazir et al. (2017), using purified bacterial DNA as a template for PCR product targeting the 16S rRNA gene using primers 27F (5-AGAGTTTGATCCTGGCTCAG-3) and 1492R (5GGTTACCTTGTTACGACTT-3). PCR products were purified by using a QIAquick PCR purification kit and sent for Sanger sequencing at the McGill University nd Genome Quebec Innovation Center. All four bacterial isolates had identical 16S rRNA gene sequences, which best match using BLAST in GenBank was a *Pseudomonas poae* sequence. The isolates were named as USP1, USP2, USP3, and USP4. The absence of antagonistic interaction between the bacterial isolates was confirmed using the cross-streak method as described by Hayat et al. (2017).

### Nematodes Extraction and Characterization

Active and free-living nematodes were extracted from the composite Saskatchewan soil following Cobb’s method (as adapted by Irshad et al., 2011). Nematodes culturing was made by inoculating the isolated nematodes on TSA (Tryptic Soy Agar) medium containing bacteria as sole source of food. The bacterial isolates used to maintain the nematodes alive were isolated from the same soil. To maintain the nematodes alive, they were collected in sterile water from 3 weeks old plates and transferred in new TSA plates. According to their morphological features, isolated nematodes belonged to two bacterial feeder families, *Rhabditidae* and *Cephalobidae*. To get single nematodes species from these mixed populations one individual egg-laden female from each of the above mentioned families was transferred to new plates. These purified nematodes populations were sterilized according to Irshad et al. (2011) to ensure that they did not harbor bacteria on their surfaces. This was achieved by a step by step process in which all the adult nematodes died, but their surface sterilized eggs remained alive and were used to generate bacterial free nematodes. TSA was used to assess nematode interaction with the four selected bacterial isolates. We used a period of 5 days to test interaction with bacteria, but in fact we monitored their preference regularly over that period. Even though nematodes react faster than this, some isolates showed very weak interactions during the first few days. Additionally, we wanted to test the interaction over a longer period, approaching the timeframe of the planned experiment. Approximately 50 individual nematodes for each of the two species isolated and purified were placed in the center of a TSA Petri plate with bacterial isolates inoculated in one quadrant each at a distance from nematodes of at least 2 cm. At regular intervals during 5 days, nematodes were counted in each zone, to quantify their feeding preferences and their interactions with the four bacterial isolates were categorized as very good, good, and not good (**Table 1**). Nematodes counts in excess of 25 individuals in a 100 μL suspension were considered as “very good interaction” with bacteria. When the counts were lower than 5 in a 100 μL suspension the interaction was scored as “not good” whereas counts between 6 and 24 individuals per 100 μL, was scored as “good.”

**Table 1:** Biochemical, ecological, and morphological characteristics of bacterial isolates from Saskatchewan wheat field soil.

Properties	USP1	USP2	USP3	USP4
Gram staining	−ve	−ve	−ve	−ve
Color	White	Yellow	Off white	Off white
Phosphate solubilization efficiency (solid media)	218	253	140	144
P-solubilization (μM) (liquid media)	2670 ± 349	466 ± 56	136 ± 14	ND
16S rRNA gene identification	*Pseudomonas poae*	*Pseudomonas poae*	*Pseudomonas poae*	*Pseudomonas poae*
Bacteria mutual interaction	+	+	+	+
Bacteria nematodes interaction^∗^	Very good	Not good	Good	Not good

*Values represent the means of six replicates. ^∗^Nematodes individuals ≥25/100 μL: very good; nematodes individuals ≤5/100 μL: not good; nematodes individuals 6–24/100 μL: good; ND, not detectable.*

### Preparation of Bacterial and Nematode Inocula

Bacterial and nematode solution was prepared for inoculation as follow: tryptic soy broth culture of bacteria USP1, USP2, USP3, and USP4 were centrifuged and the bacterial pellets were washed and re-suspended in sterilized deionized water to ensure that no P from the medium was transferred and adjusted to an optical density of 0.96. Total number of nematodes was determined by direct counting using a simple microscope. Dilutions were made and graduated Petri plates were used to count the nematodes one by one. Nematodes belonging to both families were inoculated in equivalent number, and the total number of nematodes inoculated per treatment varied from 16 to 118. This variation disappeared within a few days in the presence of plants and the numbers of nematodes observed at the harvest were orders of magnitude higher and did not correlate with the number of nematodes initially inoculated.

#### Experimental Design

Wheat seeds were surface-sterilized with H_2_O_2_ (30%) and placed on agar plates containing 2% glucose for germination. Sterile plastic containers were used for the experiment (Fisher Scientific Plantcon Plant Tissue Container, Ottawa, ON, Canada). Containers were modified by making a hole for supporting shoot growth outside.

Containers were filled with 90 ml of growth medium containing all essential elements plus agarose (Thermo Fisher Scientific, Waltham, MA, United States) as described by Irshad et al. (2011) supplemented with TCP at a rate of 4 gL^−1^ as a sole P source. Three days after germination, one seedling of wheat was transferred per container. Each inoculated container received 0.4 mL of each bacterial isolates (USP1, USP2, USP3, and USP4) or 0.4 mL of the four mixed together (USP1,2,3,4) and, if applicable, 0.1 mL of nematodes suspension (118, 15, 64, 16, and 46 nematodes, respectively) in close vicinity of the root. The experiment was conducted under controlled temperature with 16 h day and 8 h dark period with following treatments [control (plant only), USP1, USP2, USP3, USP4, USP1,2,3,4 (plant + bacteria alone/together) and USP1 + nematodes (NMT), USP2 + NMT, USP3 + NMT, USP4 + NMT, USP1,2,3,4 + NMT]. Each treatment consisted of six replicates. Water was applied to maintain constant water content in the containers, with 1 mL of autoclaved distilled water once a week. Sampling of the root associated medium (“rhizosphere”) was performed every 15 days for 2 months by coring a 1 cm hole in the vicinity of the roots. The agar plugs were placed in filter micropipette tip and centrifuged. The extract collected was used to quantify the phosphatase activity and phosphorus concentration in the medium. At the end of a 2 months period, plants were harvested by uprooting. Roots and shoots were separated to determine fresh and dry weights. The plant parts were then dried and ground for further analysis.

#### Bacterial and Nematode Abundance

At plant harvest, bacterial and nematode abundance was determined from the rhizosphere agar plug and from plate wash, respectively. DNA was extracted from the rhizosphere agar plug by using phosphate saline buffer (PSB) and quantified using a NanoDrop instrument. Total bacterial abundance was approximated by quantifying the abundance of the *phnX* gene by qPCR, using previously published primers (Bergkemper et al., 2016) on a Rotor-Gene 3000 apparatus (Corbett Life Science). This gene is present as a single copy in phosphate-solubilizing bacteria and was thus used as a proxy for bacterial abundance. No-template-controls and quantification from non-inoculated controls showed negligible quantities of *phnX* (less than 400 copies per μL). Nematodes counts were done by diluting the agar surface wash followed by direct observation and counting under the microscope.

#### Phosphorus Analysis

The water soluble P in the medium was measured during the experiment and after harvesting. Briefly, free orthophosphate concentration was measured in the medium solution or in mineralized solution using malachite green as described by Ohno and Zibilske (1991). The total root and shoot P content was measured after acid digestion of tissues as described by Irshad et al. (2012).

#### Measurement of Phosphatase Activity

Acid phosphatase activity (phosphomonoesterase) was analyzed by the modified method of Van Aarle and Plassard (2010). Briefly, the medium extraction samples were incubated with p-nitrophenyl phosphate (p-NPP) as a substrate for enzyme activity for 1 h. The reaction was terminated with 1 M NaOH and absorbance was spectrophotometrically determined at wavelength of 400 nm. Controls without medium extracted were processed in parallel to correct for background coloration. Enzymic activity is expressed as mmol p-NPP ml^−1^ h^−1^.

#### Statistical Analysis

The experiments were performed in completely randomized replicated fashion and the results were reported as mean ± standard deviation (six replicates per treatment). The differences between means were analyzed by one-way and two-way ANOVA followed by Fisher’s HSD *post hoc* test using Statistica 7.1 (StatSoft Inc., Tulsa, OK, United States). Two-way ANOVA test was done by excluding combined bacterial/nematodes treatments (using all four isolates together) to evaluate individual subspecies contribution with/without grazing. Normality was tested using the Kolmogorov–Smirnov test and data was log transformed when necessary.

### Results

#### Biochemical, Ecological, and Molecular Characteristics of Bacterial Isolates

All four screened bacterial isolates showed different colors despite the fact that they had exactly identical 16S rRNA gene sequences. Isolate USP2 was the most efficient P solubilizer compared to others when grown on solid media. On the other hand, when solubilization was measured quantitatively in liquid medium, USP1 showed the highest solubilization of insoluble P (**Table 1**). A screened isolates solubilized P in liquid media except USP4 which showed non-detectable solubilization during quantitative analysis in liquid media (**Table 1**). Interaction of bacterial isolates against each other showed positive growth relationship after 2 days of incubation on solid media. In contrast, nematodes interaction assays produced different results for the different isolates (**Table 1**). The USP1 isolate attracted more nematodes than the other isolates. USP2 and USP4 attracted nematodes the least, while USP3 showed intermediate results (**Table 1**). In all treatments, nematodes increased in numbers during the 2 months of the experiment, indicating that they were feeding on the inoculated bacteria.

#### Acid Phosphatase Activity

**FIGURE 1:**
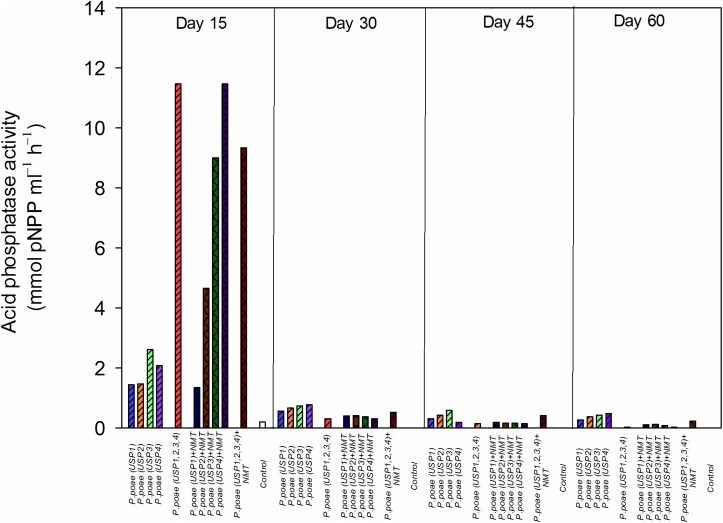
Acid phosphatase activity in the rhizosphere of wheat seedlings after 15, 30, 45 or 60 days under the different treatments. Bars represent the means of six replicates.

The acid phosphatase quantity produced in rhizosphere was detected in significant amounts only at day 15. The highest concentration of phosphatase was produced by treatments USP3 + NMT, USP4 + NMT, USP1,2,3,4, and USP1,2,3,4 + NMT and ranged from 7 to 12 mmol p-NPP mL**^−^**^1^ h**^−^**^1^ (**Figure 1**). All other treatments had significantly lower phosphatase activity (≤3 mmol p-NPP mL**^−^**^1^ h**^−^**^1^) except for USP1 + NMT which showed intermediate values (**Figure 1**). Results of two-way ANOVA which was performed by excluding combined bacterial/nematodes treatments (all four strains together) demonstrated that presence of nematodes significantly increased the amount of phosphatase at day 15 compared to individual inoculated bacteria (**Figure 1** and **Table 3**).

### Medium Free Phosphorus

**FIGURE 2:**
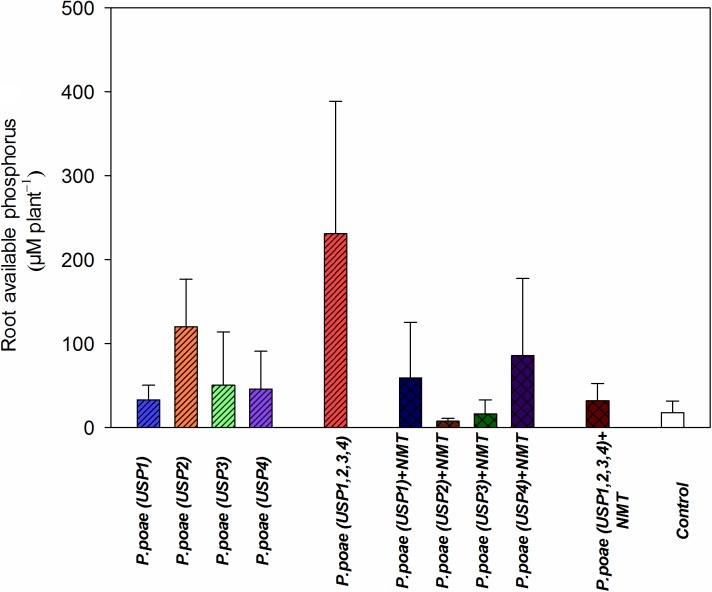
Available phosphorus concentration in the rhizosphere of wheat seedlings after 60 days under different treatments. Bars represent the means of six replicates with SD (standard deviations).

The different treatments resulted in variable amounts of P liberated from insoluble source at day 60 (**Figure 2**). Treatment USP1,2,3,4 showed the highest average release (>380 μM) of free P per plant culture container. All other treatments were not significantly different from each other, and when compared to the non-inoculated controls (**Figure 2** and **Table 3**). Addition of nematodes reduced medium P status, but this trend was not significant (**Figure 2** and **Table 3**).

### Plant Dry Biomass

**FIGURE 3:**
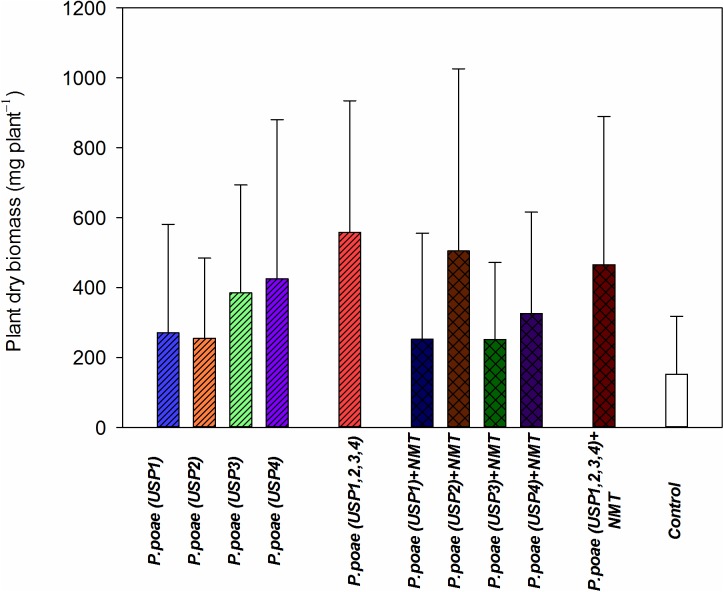
Plant dry biomass of wheat seedlings after 60 days of growth under different treatments. Bars represent the means of six replicates with SD (standard deviations).

Diverse responses were found for plant dry biomass between the different treatments. All treatments showed a trend toward increased plant biomass as compared to the controls (**Figure 3**). When combined together, USP1,2,3,4 resulted in a synergistic responses both when inoculated with nematodes or not (**Figure 3**). Results of the two-way ANOVA (**Table 3**) showed a significant effect interaction between bacterial isolates and nematode presence.

### Abundance of the *phnX* Gene

**FIGURE 4:**
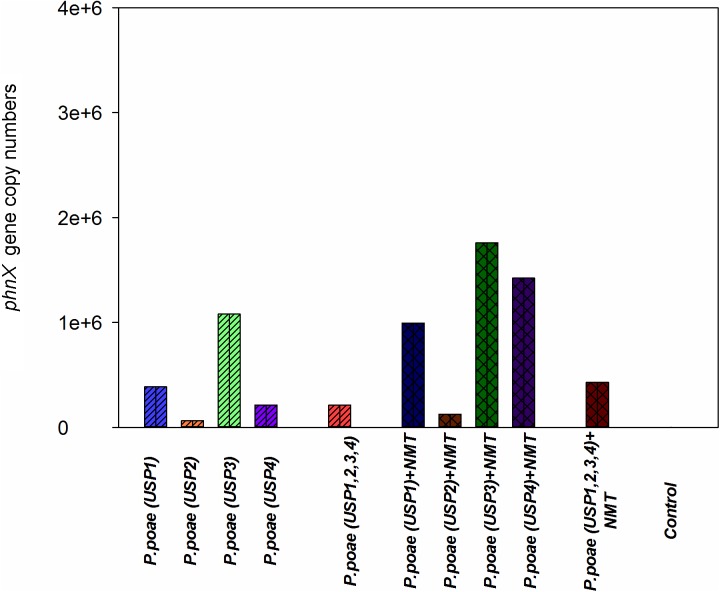
Abundance of the *phnX* gene measured by qPCR in the rhizosphere of wheat seedlings after 60 days of growth under different treatments.

qPCR quantification of the *phnX* gene was used as a proxy for the abundance of the inoculated bacteria as all four isolates were PCR positive for this gene, it is a key gene for P-mineralization and is present in a single copy. Four treatments (USP3, USP1 + NMT, USP3 + NMT, and USP4 + NMT) resulted in higher gene copy number as compared to the un-inoculated control and most other treatments. Surprisingly, most of these treatments included inoculation with bacterial grazing nematodes. All other treatments with or without nematodes were not different from each other or from the control (**Figure 4**). Two-way ANOVA demonstrated that the presence of nematodes significantly increased bacterial abundance and that there were significant differences between the bacterial isolates (**Table 3**).

### Total Plant P Concentration

**FIGURE 5:**
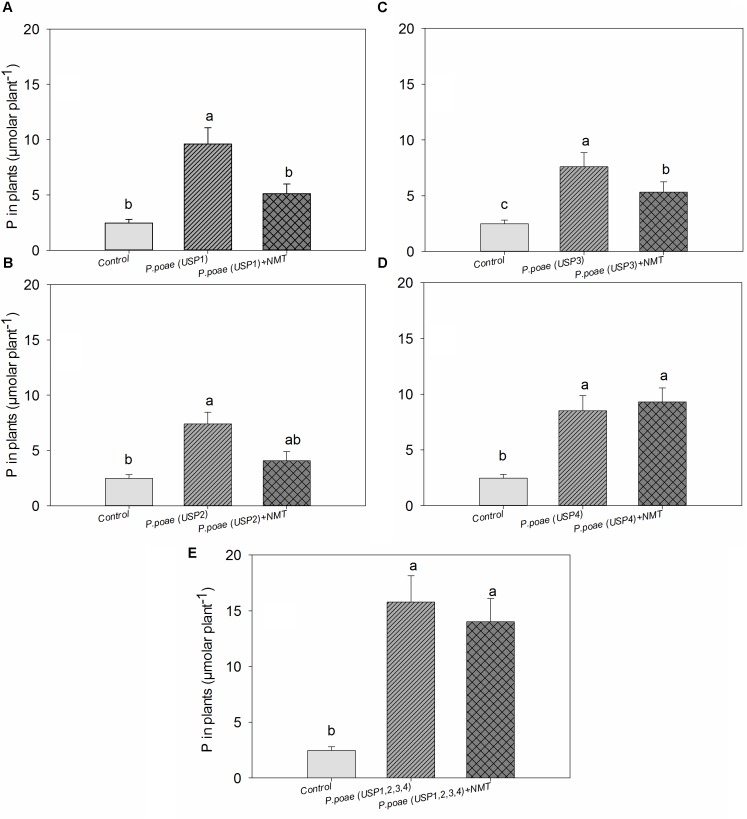
Total phosphorus concentration in wheat seedlings after 60 days of growth. **(A–E)** Represents the results for the four different strains and the mixture of all four strains. Bars represent the means of six replicates with SD (standard deviations). Different lowercase letters indicate significantly different means between the treatments according to Tukeys’s HSD *post hoc* test at *P*< 0.05.

Plant total P concentration showed significant differences between the bacterial isolates (**Figure 5**). All bacterial isolates increased plant P concentration significantly as compared to the uninoculated control. Nematodes grazing significantly decreased plant P concentration for isolates USP1, USP2, and USP3. The combined treatment where all bacterial isolates were inoculated resulted in a significant increase in plant P concentration as compared to control, and this difference remained when nematodes were also inoculated (**Figure 5**). Two-way ANOVA test showed a significant effect of both individual isolates and nematodes grazing separately but the interaction term was found non-significant (**Table 3**).

### Effects of Multiple Closely Related Strains

In order to compare the effects of microdiversity and nematode grazing, single species treatments were lumped together for analysis and compared to the treatment using all four isolates together, with and without nematodes (**Table 2**). All isolates together with or without nematodes showed significantly more phosphatase activity when compared to individual isolates used alone with or without nematodes. Significantly lower abundance of the *phnX* gene was observed in the single isolate treatment without nematodes as compared to the single isolate treatment with nematodes (**Table 2**). The four isolates treatments had intermediate abundance.

**Table 2:** Comparison of the effects of nematode grazing and bacterial diversity on phosphatase, medium free P, plant P concentration, and *phnX* gene abundance for wheat seedlings grown for 60 days.

Treatments/parameters	Phosphatase activity (mmol p-NPP ml^−1^ h^−1^)	Medium free P (μM plant^−1^)	Plant P concentration (μM plant^−1^)	*phnX* gene abundance (gene copies plant^−1^)
Bacterial single isolate	1.97 c	112 b	7 b	721,264 b
Bacterial single isolate + NMT	4.23 b	82 b	5 b	3,241,657 a
All bacterial isolates	8.45 a	342 a	16 a	424,991 ab
All bacterial isolates + NMT	7.47 a	46 b	14 a	858,864 ab

*p*-values	≤0.01	≤0.01	≤0.01	≤0.05
*F*-ratio	27.21	12.73	18.67	4.54

*df*	3	3	3	3

*Values represent the means of 4–6 replicates. Different lowercase letters indicate significantly different means between the treatments according to Tukeys’s HSD post hoc test at P < 0.05. Single bacterial isolate: average values when the four isolates (USP1,2,3,4) were used alone. All bacterial isolates: average values when all isolates (USP1,2,3,4) were combined and used together.*

Medium free P was significantly higher when all bacterial isolates were inoculated without nematode grazing as compared to all other treatments (**Table 2**). Plant P concentrations was not entirely coherent with the medium free P concentrations, with the individual isolate treatments showing significantly lower P concentrations (5–7 μM plant**^−^**^1^) than the multi-isolates treatment, irrespective of the inoculation with nematodes.

## Discussion

Extensive use of phosphorus-based chemical fertilizers is one of the major cost associated with cropping. But due to its reactive nature in soil, less than 5% of applied P becomes bioavailable for crops (Brady and Weil, 2008; Bulgarelli et al., 2013). Therefore, the use of microbial inoculants such as bacteria to increase P availability for plant growth has been gaining traction. Many bacterial genera, including *Pseudomonas*, were shown to be able to perform calcium-bound P solubilization and organic P-mineralization (Parani and Saha, 2012). Here, we explored the variation in the P solubilization capacity among different, closely related isolates identified as *Pseudomonas poae*. Although our four isolates had identical 16S rRNA gene sequences, they exhibited varying capacities for P solubilization and free P in liquid media ranged from non-detectable up to 2670 μM (**Table 1**). Similar evidence was given by Illmer and Schinner (1992), where *Pseudomonas* sp. varied in solubilization capability from 52 to 156 mgL**^−^**^1^ when grown on Ca_3_(PO_4_)_2_ in liquid cultures. Singh et al. (2014) reported different P-solubilization capacities among different strains belonging to same *Advenella* species.

Inoculation of a single isolate providing P to plant can have beneficial effects on plant growth and yields (Selvakumar et al., 2009; Prasad et al., 2012; Aftab et al., 2014), but mixed species inocula have often been shown to be more efficient. For instance, Braz and Nahas (2012) found that organic acid production and phosphate solubilization were greater in co-cultures of *Aspergillus niger* and *Burkholderia cepacia* than when they were growing alone. Here, we reported that synergistic interactions among our four closely related *Pseudomonas poae* isolates increased free P concentrations by more than 300 μM. Rodriguez and Fraga (1999) showed the synergistic effects of combining multiple phosphate-solubilizing bacteria on P status, but it is the first time that this synergistic effect is shown for very closely related isolates. Because of technical limitations, the importance of subspecies level bacterial diversity for nutrient cycling has been widely overlooked, but could have a crucial importance for agriculture.

The differences between closely related isolates in term of P-solubilization were also visible in term of plant P uptake when the isolates were applied to the wheat rhizosphere (**Figure 5**). Similar variations in P uptake by wheat seedlings were reported when inoculated with different *Pseudomonas* strains (Zabihi et al., 2011). However, in our study, the most efficient solubilizers *in vitro* were not necessarily the best at improving plant P uptake (**Table 1** vs. **Figure 5**). Similarly, closely related *Advenella* sp. isolates did not show congruent relative efficiency when compared alone or in the presence of plant roots (Singh et al., 2014). It has indeed been observed that P solubilization capacity of bacteria in liquid media could be different when compared to P solubilization capacity in the plant rhizosphere (Gyaneshwar et al., 2002; Irshad et al., 2011).

The presence of bacterivorous nematodes also modified the bacterial capacity to solubilize P and to make it available to the wheat plant. For instance, the phosphatase activity was significantly enhanced by the presence of nematodes (**Figure 1** and **Table 2**), in line with recent work that showed an increased alkaline phosphatase activity when bacteria were grazed by nematodes (Jiang et al., 2017). Nematodes presence also had a generally positive effect on the other variables measured such as rhizosphere available P, bacterial abundance, and plant P concentrations, but this often varied between our different *Pseudomonas poae* isolates. For instance, we observed a net twofold to fourfold increase in the available rhizosphere P when all bacteria were combined, but not when nematodes were added (**Figure 2**). This is in line with Gebremikael et al. (2016) that reported no significant effects of nematodes on water soluble inorganic P concentration even though plant available P did significantly increase. Plant dry biomass measures were variable and indicated a mixed effect of all inoculations, with some negative effects of nematodes. Indeed, individual isolates increased dry plant biomass compared uninoculated plants but mostly without grazing nematodes (**Figure 3** and **Table 3**). These biomass increases by bacterial inoculations disagree with previous results obtained by Singh et al. (2014) where they inoculated Indian mustard with different bacteria of same species without any clear effect. The presence of nematodes also significantly increased the abundance of our bacterial isolates (**Figure 4**), in line with previous reports for maize and pine (Irshad et al., 2011, 2012; Jiang et al., 2017). However, here again, this was not constant across all our closely related isolates. Plant accumulated P was found here to be most strongly enhanced when all four *Pseudomonas poae* isolates were combined together and subjected to nematodes grazing (**Figure 5**). In fact, nematode addition with a single *P. poae* isolate had inconstant effects on P availability and plant P content, in sharp contrast with previous reports (Irshad et al., 2012; Gebremikael et al., 2016). The differences observed here between the efficiency of the different isolates to make P available in the presence of nematodes might be due to feeding preferences of nematodes, highlighting their important role in shaping the bacterial community and thereby modulating P availability. As a potential explanation for this differential grazing, Jiang et al. (2017) reported that different bacteria use contrasting physical and chemical means to avoid nematode predation. As these interactions might reduce the potential benefit of utilizing P-solubilizing bacteria, it appears important to test the interaction of each individual bacteria and multi-species inoculums with organisms from higher trophic levels even if the bacterial isolates are closely related. Some of the differences observed in the effects of nematodes on the capacity of bacteria to make P available could also be due to the fact that the number of nematodes presents at the beginning and the end of the experiment varied substantially between treatments.

**Table 3:** Comparison of the effects of nematode grazing and bacterial isolates on phosphatase, medium free P, plant P concentration, *phnX* gene abundance, and plant biomass for wheat seedlings grown for 60 days.

	Treatments	Phosphatase activity (mmol p-NPP ml^−1^ h^−1^)	Medium free P (μM plant^−1^)	Plant P concentration (μM plant^−1^)	*phnX* gene abundance (gene copies plant^−1^)	*Plant biomass* (mg plant^−1^)
Single bacterial isolates	*P. poae* (USP1)	2.05*cd*	45.27*a*	9.60*a*	772,670 ab	490 cd

	*P. poae* (USP2)	1.67*d*	159.89*a*	6.29*bcd*	127,145 b	417 d
	*P. poae* (USP3)	2.60*cd*	95.06*a*	7.60*abc*	2,158,246 ab	603 bc
	*P. poae* (USP4)	1.54*d*	342.40*a*	9.35*ab*	427,209 ab	747 ab
Single bacterial isolates with nematodes	*P. poae* (USP1)+ Nem	2.95*c*	105.80*a*	5.12*cd*	1,989,040 ab	467 cd

	*P. poae* (USP2)+ Nem	1.68*d*	9.80*a*	3.93*d*	253,570 ab	873 a
	*P. poae* (USP3)+ Nem	4.64*b*	27.70*a*	5.31*cd*	3,521,250 a	407 d
	*P. poae* (USP4)+ Nem	7.64*a*	46.25*a*	9.29*ab*	2,844,678 ab	531 cd

Bacterial isolates		<0.001	0.199	0.006	0.001	0.004
Nematodes presence		<0.001	0.454	0.004	0.001	0.892
Bacterial isolates x Nematodes presence		<0.001	0.795	0.222	0.788	<0.001

*Values represent the mean of 4–6 replicates. In the bottom of the table, p-vales for two-way ANOVA are given. Different lowercase letters indicate significantly different means between the treatments according to Tukeys’s HSD post hoc test at P < 0.05.*

## Conclusion

We have shown here that even among very closely related *Pseudomonas* isolates, the potential to improve wheat growth and P nutrition varies greatly. The best results were obtained when mixing the four isolates, suggesting that consortia of closely related species might be useful to increase processes of interest. In contrast to previous reports, under our experimental settings the effect of nematodes grazing was not always positive for plant P concentrations, but grazing did increase phosphatase enzyme and bacterial abundance. Our results would have to be confirmed using robust field experiments, but the idea that bacterial diversity at the subspecies level could play a role in important soil processes is very enticing and particularly novel. It also questions the appropriateness of tools that lack a subspecies resolution to study key soil processes such as P-solubilization.

## Author Contributions

UI planned and performed the research, carried out data analysis, and was lead author for the manuscript. EY helped with planning and research, editing the manuscript, and secured the funding for the project.

## Conflict of Interest Statement

The authors declare that the research was conducted in the absence of any commercial or financial relationships that could be construed as a potential conflict of interest.
